# Effects of Newtonian gravitational self-interaction in harmonically trapped quantum systems

**DOI:** 10.1038/srep30840

**Published:** 2016-08-04

**Authors:** André Großardt, James Bateman, Hendrik Ulbricht, Angelo Bassi

**Affiliations:** 1 Department of Physics, University of Trieste, 34151 Miramare-Trieste, Italy; 2 Istituto Nazionale di Fisica Nucleare, Sezione di Trieste, Via Valerio 2, 34127 Trieste, Italy; 3 School of Physics and Astronomy, University of Southampton, SO17 1BJ, United Kingdom; 4 Department of Physics, College of Science, Swansea University, Swansea SA2 8PP, United Kingdom

## Abstract

The Schrödinger–Newton equation has gained attention in the recent past as a nonlinear modification of the Schrödinger equation due to a gravitational self-interaction. Such a modification is expected from a fundamentally semi-classical theory of gravity and can, therefore, be considered a test case for the necessity of the quantisation of the gravitational field. Here we provide a thorough study of the effects of the Schrödinger–Newton equation for a micron-sized sphere trapped in a harmonic oscillator potential. We discuss both the effect on the energy eigenstates and the dynamical behaviour of squeezed states, covering the experimentally relevant parameter regimes.

The interaction of nonrelativistic quantum matter with an *external* gravitational field has been experimentally established by the famous COW experiment^1^. However, the question whether gravity is fundamentally a quantum theory resembling the other fields, or something different, is still open. There is no unambiguous answer to the question how quantum matter sources the gravitational field. While the standard approach in regard to the great success of quantum field theory is to quantise the gravitational field along similar lines, there is no experimental evidence, nor a strict theoretical necessity, to date, that the gravitational field must be quantised^2^,^3^,^4^.

Taking the possibility of a fundamentally classical description of space-time into account, the most natural way to describe the interaction of quantum matter with such a classical space-time within the framework of general relativity is provided by the semi-classical Einstein equations,


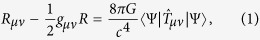


i.e. Einstein’s field equations where the energy-momentum tensor is replaced by the expectation value of a corresponding quantum operator in some quantum state Ψ; a theory that was already suggested in the 1960s by Møller^5^ and Rosenfeld^2^. (Although it is often claimed that a fundamentally semi-classical theory of gravity was ruled out by experiment^6^, the arguments against such a theory are inconclusive^3^,^4^,^7^. It needs to be stressed that, different than in other situations where semi-classical gravity is considered as an effective limit of some underlying quantum theory of gravity^8^, in this approach equation (1) is taken as fundamental, at least at the microscopic level of single atoms).

In the nonrelativistic limit, such a fundamentally semi-classical theory of gravity adds a nonlinear potential term to the Schrödinger equation^7^,^9^. The resulting equation is known as the *Schrödinger*–*Newton equation*. For a multi-particle system, it reads


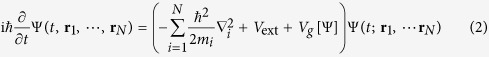



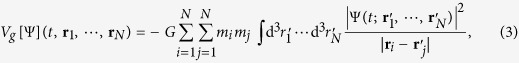


where Ψ is the *N*-particle wave-function, and *V*_*g*_ is the gravitational interaction. The Schrödinger–Newton equation becomes nonlinear due to the dependence of *V*_*g*_ on the absolute-value squared of the wave-function. An intuitive way of looking at this equation is that the probability density, |Ψ|^2^, acts like a mass density generating a Newtonian gravitational potential, which then appears in the Schrödinger equation in the usual way. *V*_ext_ is an external potential, which will be a quadratic, i.e. harmonic oscillator, potential here.

Equation (2) was first considered by Diósi^10^ as a model for wave-function localisation. Because of its derivability from semi-classical gravity, it was suggested that the Schrödinger–Newton equation can provide evidence for or against the necessity to quantise the gravitational field^11^. The original subject of such a test were heavy molecules in interferometry experiments^12^ for which the Schrödinger–Newton equation predicts inhibitions of the dispersion of the centre-of-mass wave-function^13^,^14^,^15^,^16^,^17^. Although the parameter regime where this effect shows up is much closer to the scope of current experiments than any quantum gravity effect studied so far, the required masses are still several orders of magnitude above what is currently feasible.

An alternative test of the Schrödinger–Newton equation, using macroscopic quantum systems in a harmonic trap potential, is given by Yang *et al*.^18^, who show that that the Schrödinger–Newton dynamics lead to a phase difference between the external and internal oscillations of a squeezed Gaussian state. Here, we complement this proposal by a more general analysis of effects of the Schrödinger–Newton equation on harmonically trapped quantum systems, going beyond the limit of narrow wave-functions and considering also the regime where the width of the wave-function becomes comparable to the localisation length of the atoms in the considered microsphere. In addition to the dynamical effects, we also discuss the gravitational perturbation of the spectrum of the stationary energy eigenstates.

While we will find that the dynamical effect on the internal structure of a squeezed state is indeed strongest in the limit of a narrow wave-function, as it has been studied by Yang *et al*.^18^, the intermediate regime is the most suitable to observe effects in the energy spectrum. These turn out to be of comparable order of magnitude as the dynamical effects. However, their observation requires slightly smaller masses and, more importantly, there is no necessity to create a squeezed state, which makes it an interesting alternative for feasible experimental tests. A possible experimental setup, achievable with existing technology, has been proposed elsewhere^19^.

We present the Hamiltonian for the trapped system with Newtonian self-gravitational interaction in the second section. We derive an approximation for the gravitational interaction inside a crystalline spherical many-particle system and discuss the reduction of the three-dimensional equation to a one-dimensional Schrödinger–Newton equation, which is the basis for the discussion thereafter. In the third section, we study the effects of the Schrödinger–Newton interaction on the energy spectrum. We discuss the limiting cases of a narrow and wide wave-function, as well as the intermediate regime. In the fourth section, the dynamical behaviour of a squeezed Gaussian state is derived, recovering the results by Yang *et al*.^18^ Their results are extended to the regime of finite, non-narrow wave-function sizes. Finally, our results and the prospects for experimental tests of the Schrödinger–Newton equation are summarised in the Discussion section.

## Hamiltonian of a self-gravitating trapped sphere

Consider a three-dimensional Hamiltonian of a self-gravitating quantum system in an external potential:


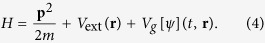


The coordinates are written as **r** = (*x*, *y*, *z*). We will specify the external potential *V*_ext_ later.

The Hamiltonian (4) is supposed to describe the centre-of-mass of a many-particle system. The gravitational potential, which is a function of all particle coordinates, does, however, not separate into centre-of-mass and relative coordinates exactly. Such a separation can only be achieved within a suitable Born–Oppenheimer-type approximation^16^. The multi-particle gravitational potential can then be reduced to


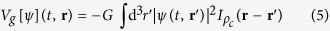



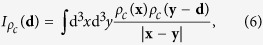


where *ψ* is the centre-of-mass wave-function, **r** is the centre-of-mass coordinate, and *ρ*_*c*_ is the mass density relative to the centre of mass. For a lump of matter, i.e. a molecule, of *N* atoms which is described by a stationary relative wave-function *χ*, *ρ*_*c*_ is given as the sum of the marginal distributions for all but one atoms (the distribution of the *N*-th particle is given by the centre-of-mass wave-function and can be neglected if *N* is sufficiently large):


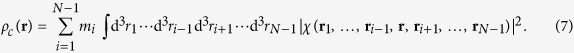



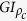
 is simply the gravitational potential energy between the mass distribution described by *ρ*_*c*_ and the same mass distribution, shifted by **d**. For a homogeneous, spherical mass distribution with radius *R* it is given by^20^


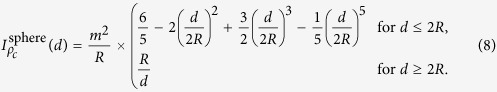


Given a solution *ψ*^(0)^ of the free Schrödinger equation (without the gravitational potential *V*_*g*_), switching on the state dependent gravitational potential (5) will distort both the energy expectation value and the shape of the solution. To first order in the gravitational constant *G*, the correction to the Schrödinger evolution due to the nonlinear Schrödinger–Newton gravitational potential term can be obtained in a perturbation expansion. For this purpose, we make the ansatz


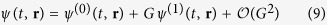


for the wave-function. Now note that the perturbation *V*_*g*_ of the Hamiltonian can be expanded as


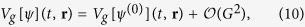


where the first term is already 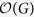. 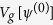 is just a linear correction to the Hamiltonian, which is time-independent for a stationary state *ψ*^(0)^. The Hamiltonian (4) then takes the linear form


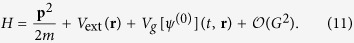


This is a good approximation as long as the gravitational interaction is considered to be weak, and therefore the difference in the wave-function between the solutions of the unperturbed Schrödinger equation and those of the full Schrödinger–Newton equation is small.

The potential (5) can be considerably simplified in the limits where the wave-function is very narrow or very wide. Provided that the spatial centre-of-mass wave-function is wide compared to the extent of the considered many-particle system, the mass distribution within the system plays no important role, and the gravitational potential is approximately the same as in the one-particle case, namely^16^


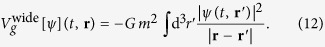


Consider, on the other hand, the case where the spatial centre-of-mass wave-function is narrow compared to the extent of the many-particle system, or—to be more precise—where 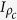 does not vary too much over the width of the centre-of-mass wave-function. In this case the potential (5) can be expanded in a Taylor series in |**r** − **r**′| up to second order, yielding^16^,^18^


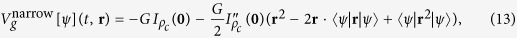



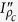
 denoting the Hessian of 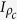.

If the mass is assumed to be distributed homogeneously over a sphere of radius *R*, and therefore the function 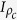 takes the form (8), then the potential is^16^


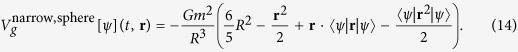


However, as pointed out by Yang *et al*.^18^, a realistic microsphere has a crystalline substructure, which must be taken into account if the wave-function is narrow enough to probe the atomic regime.

### Crystalline substructure

**Figure 1:**
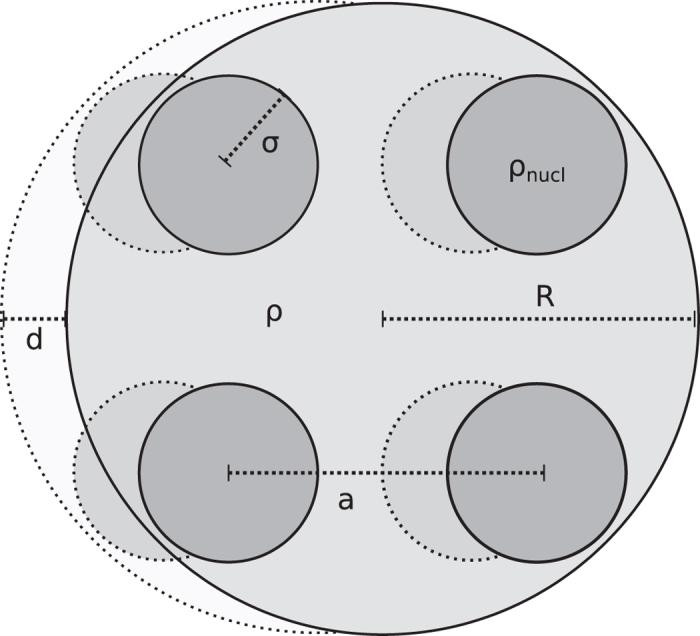
Schematic picture of how the function 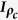 is determined for a sphere of atoms in a cubic lattice.

A more realistic mass distribution should account for the fact that most of the mass in a crystalline structure is well-localised around the positions of the nuclei. 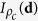 then represents the gravitational interaction of a grid of *N* atoms with an identical grid, shifted by distance **d**. We model the quantum system as a sphere of radius *R*, within which the atoms are homogeneous spheres of radius *σ*, as depicted in Fig. 1. There are two contributions to 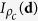:

1. The self-energy of each atom with its own “copy” which, approximately, can be modelled as the gravitational self-interaction of a sphere of radius *σ* with mass *m*/*N*, hence


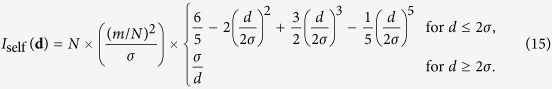


2. The mutual interaction of each atom with all *N* − 1 other atoms, which is the Riemann sum for the integral (6) for the full sphere of radius *R*, if the sphere is split into *N* sub-areas of volume *a*^3^, hence


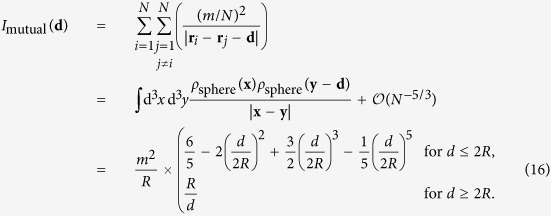


Therefore, for large *N*, the total function 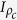 for a crystalline sphere is


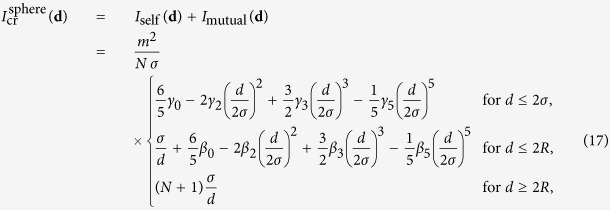


with


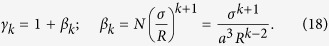


Note that the expansion (13) is still valid in the limit of a narrow wave-function, which now means that the width of the wave-function is small compared to the atomic radius *σ*. Making use of the fact that *γ*_2_ ≈ 1 for 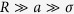, the corresponding gravitational potential is


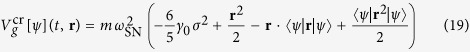


with


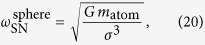


where *m*_atom_ is the mass of a single atom in the crystal. This potential has been used by Yang *et al*.^18^ to describe the behaviour of a narrow squeezed coherent state in a harmonic trap. Since it is quadratic in **r**, a Gaussian state will remain Gaussian^17^,^18^, but there will be a gravitational contribution to the coupling constant. We come back to this below, in the section about the dynamics of a squeezed state.

If the atoms are, more realistically, modelled by Gaussian matter distributions^18^,


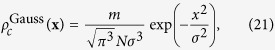


one can see with equation (6) that the self-interaction part of the function *I*_cr_ takes the form^20^


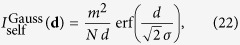


where erf is the Gauss error function. The total *I*_cr_ then is


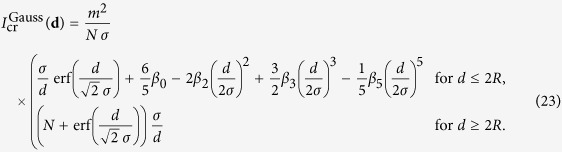


For the Gaussian matter distribution, the gravitational potential of a narrow wave-function is of the same shape (19), but with the frequency *ω*_SN_ and *γ*_0_ replaced by


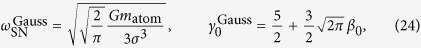


where we assumed 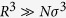. This result should in principle agree with equation (14) in the work by Yang *et al*.^18^, provided that their 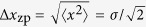. However, we find a factor of 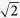 difference compared to their expression for 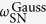.

It should be remarked that, while the splitting in cubes of volume *a*^3^ in the derivation provided here seems to imply the requirement of a simple cubic crystal structure, the result is actually independent of the type of the present crystal structure. Even a non-crystalline, amorphous substructure will still exhibit the behaviour described here, as long as the localisation length *σ* of the atoms is small compared to the average distance *a* between the atoms.

### Reduction to one dimension

An approximate one-dimensional version of the Schrödinger–Newton equation can be obtained in the case where the shape of the external potential is such that the wave-function will be narrow in the two remaining dimensions. In this case, where the wave-function satisfies approximately


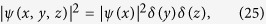


the gravitational potential (5) takes the form


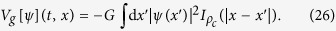



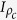
 is an even function by definition for any matter distribution *ρ*_*c*_, hence the absolute value in the argument of 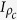. The dependence of the argument on *y* and *z* can be neglected, because the parts where *y* or *z* is substantially different from zero do not contribute in the Schrödinger equation after multiplication with the wave-function. Substituting *d* = |*x* − *x*′| the potential can be rewritten as


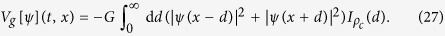


The functions (17) and (23) can now be applied to this one-dimensional potential without any changes, and the Schrödinger equation separates and yields the one-dimensional equation


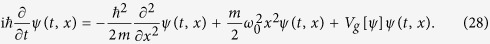


Note that we assume now that the external potential is quadratic with trap frequency *ω*_0_ in *x*-direction, while the shape of the external potential in *y*- and *z*-direction does not play a role, as long as the wave-function will be narrow.

The one-dimensional potential (27) still has the corresponding limits


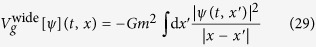



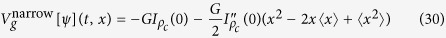


for a wide (in *x*-direction) and narrow wave-function, respectively.

## Gravitational effects on the energy spectrum

Without the gravitational potential *V*_*g*_, the Schrödinger equation (28) has the well known energy eigenstates


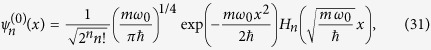


where the Hermite polynomials *H*_*n*_ are defined by


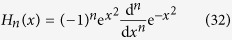


and the corresponding energy eigenvalues are


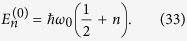


As long as one is only concerned with stationary solutions, one can perform a first-order perturbation calculation to obtain the energy correction coming from the gravitational potential. In the quadratic narrow wave-function approximation (19) we immediately get the energy correction


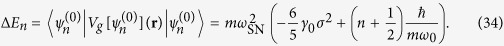


In this approximation, the first term is just a constant shift of all energy levels, while the second term changes the spectral transition energies proportionally to 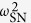. The transition energies,


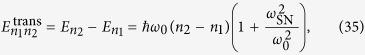


are, however, still degenerate, i.e. they depend only on the difference (*n*_2_ − *n*_1_), and not on *n*_1_ and *n*_2_ alone. This degeneracy is removed if the higher order terms in the gravitational potential are taken into account, leading to a fine-structure of the spectral lines. Note that this fine-structure of the harmonic oscillator is of a different nature than the well-known fine-structure of atomic spectra. While in the latter there is a degeneracy of the actual energy eigenvalues, that is removed by additional interaction terms, the one-dimensional harmonic oscillator has an infinite number of *non-degenerate* energy eigenstates whose energy eigenvalues are shifted due to the Schrödinger–Newton potential. The degeneracy here is in the transition spectrum, where transition energies between eigenstates depend on the difference (*n*_2_ − *n*_1_) only. This is the degeneracy that is removed by the Schrödinger–Newton term.

To arrive at equation (34) we made use of the approximation (10). As mentioned before, in this case the gravitational potential is just a linear correction and the energy shift can be calculated in ordinary perturbation theory. Maintaining this approximation, but now using the full gravitational potential (26) instead of the quadratic approximation for narrow wave-functions, one obtains:


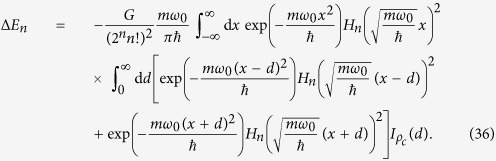


Introducing the dimensionless variables


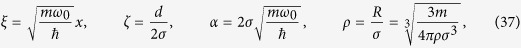


we get


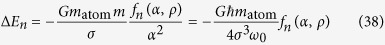


with


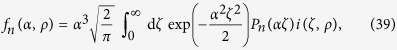


the even polynomials


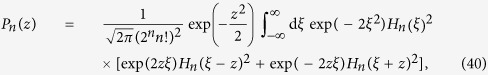


and the matter distribution functions


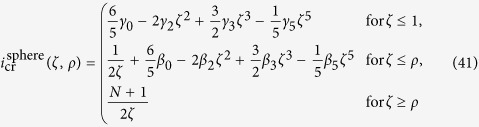


for spherical atoms, and


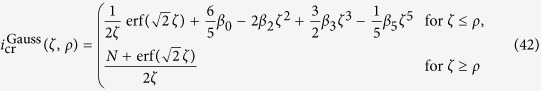


for a Gaussian distribution of the atomic matter density, respectively. The polynomials *P*_*n*_ can be solved analytically, and so can the functions *f*_*n*_, at least in principle. We obtained *P*_*n*_ for small *n* up to *n* = 14 using *Mathematica*. The time needed for the evaluation increases, however, exponentially with *n*. *f*_*n*_ can be integrated analytically as well, in terms of integral functions. For realistic physical parameters it is nevertheless necessary to omit the constant contribution 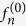 (see text below for definition) in a numerical evaluation of the transition energies, since otherwise the subtraction of two comparable, large numbers from each other would lead to a substantial loss of numerical accuracy. The transition energies can then be calculated as


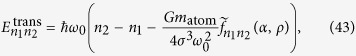



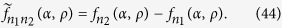


### Narrow wave-functions

In the limit of large values for *α*, i.e. narrow wave-functions, we can write


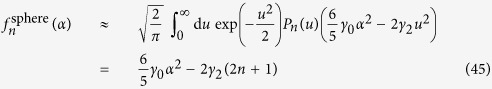


for the spherical atomic mass distribution, and


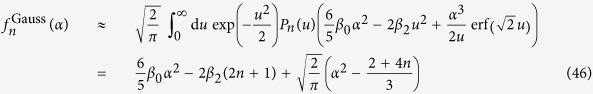


for the Gaussian distribution, and hence


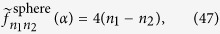



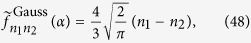


where we used that *γ*_2_ ≈ 1 and *β*_2_ ≈ 0 for *R* ≫ *a* ≫ *σ*. This yields exactly the previous results (35) with the frequencies (20) and (24).

### Intermediate wave-functions

Now we want to go beyond the quadratic approximation for the potential, to see how the gravitational interaction removes the degeneracy of the transition energies. For this, we consider the intermediate regime where *α* is of the order of unity. We are, again, interested in the case where *N* ≫ 1 and *R* ≫ *a* ≫ *σ*. In this case, we have *γ*_0_ ≈ *β*_0_, 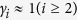, and *ρ* ≫ 1. We can then write for the spherical atomic mass distribution


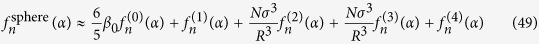


with


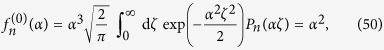



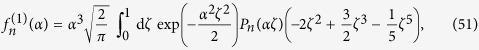



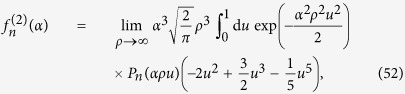



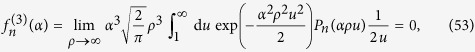



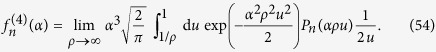


**Figure 2:**
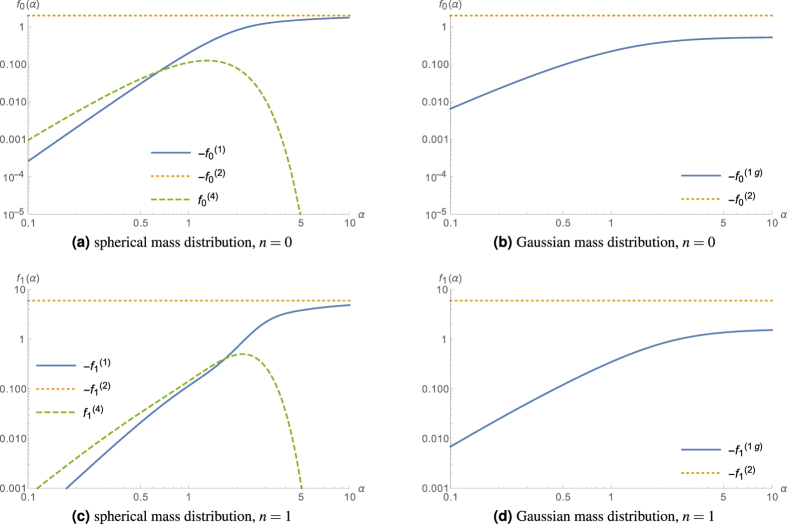
Comparison of the different terms contributing to *f*_*n*_(*α*) for the spherical and Gaussian atomic mass distribution, for the ground state as well as the first excited state.

These functions become *ρ*-independent by taking only the zeroth order of the expansion around infinity. 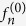 is an *n*-independent term, which is large but does not contribute to the transition energies. 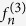 can be neglected since it goes to zero exponentially as *ρ* becomes large. The remaining terms, 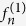, 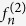, and 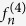, are of comparable size, cf. Fig. 2. 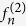 however enters into the full function *f*_*n*_ with the small pre-factor *Nσ*^3^/*R*^3^ (which is, e.g., less than 10^−4^ for silicon and less than 10^−5^ for osmium at a few Kelvin), and therefore can be neglected as well, allowing for a *ρ*-independent approximation for *f*_*n*_. Figure 2 shows that for *α* > 1, 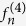 is also negligible compared to 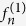. We nevertheless use both functions to calculate the transition energies with


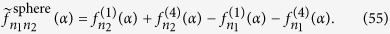


For the Gaussian atomic mass distribution one obtains instead


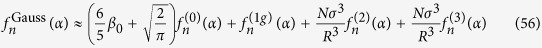


with


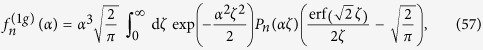


were we accounted for the *n*-independent, 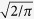-proportional, contribution of 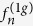 in 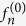. With the same arguments as before, this results in


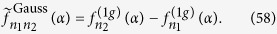


**Figure 3:**
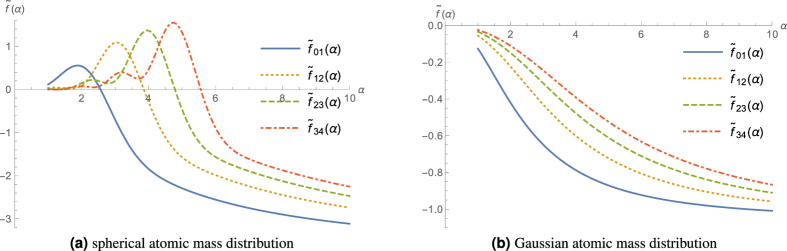
The coefficient function 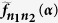 for the spherical and Gaussian atomic mass distribution.

In Fig. 3 these functions are plotted for the lowest four transitions with Δ*n* = 1, for both the spherical and Gaussian atomic mass distribution. One can see how the degeneracy is removed, and there is a split of 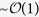 for the spherical, and 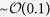 for the Gaussian distribution, respectively. In the limit of an infinitesimally narrow wave-function, i.e. 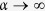, all these functions will converge against the same value, in agreement with equation (35).

The order of magnitude of the split of the spectral lines belonging to the same Δ*n* is given by the pre-factor in equation (43). Taking, e.g., silicon^21^ at a few Kelvin with *m*_atom_ = 28 u and *σ* ≈ 7.0 × 10^−12^ m, we get


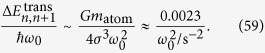


**Figure 4:**
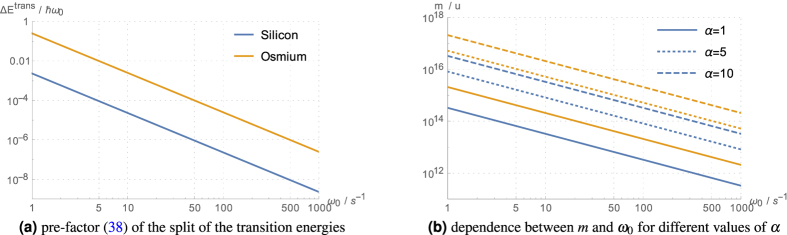
The first plot shows the dependence of the pre-factor (59) of the split in transition energy on the trap frequency *ω*_0_. The second plot shows the necessary mass for *α*-values of 1, 5, and 10, respectively, for these frequencies *ω*_0_. We used the values for silicon (in blue) and osmium (in orange) as given in the text.

The best value can be obtained for osmium^22^ with *m*_atom_ = 190 u and *σ* ≈ 2.8 × 10^−12^ m, where the above pre-factor is two orders of magnitude larger. Figure 4 shows the dependence of this pre-factor on the trap frequency, and the corresponding masses for different values of *α*.

Qualitatively, the same effect can be expected from a three-dimensional harmonic oscillator, in situations where the wave-function has comparable width in all directions, although the situation gets more complicated when transitions are allowed in all three dimensions with different frequencies. We study the simpler case of an axially symmetric state which is excited in only the longitudinal direction in the Supplementary Information A.

### Wide wave-functions

In the limit of a wide wave-function, *α* → 0 and 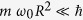, the dominant contribution comes from the function 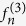, yielding


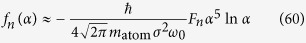


with the *n*-dependent pre-factors


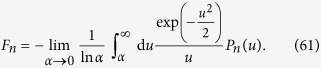


The first six values for the pre-factors are


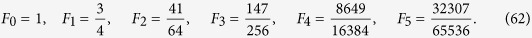


**Figure 5:**
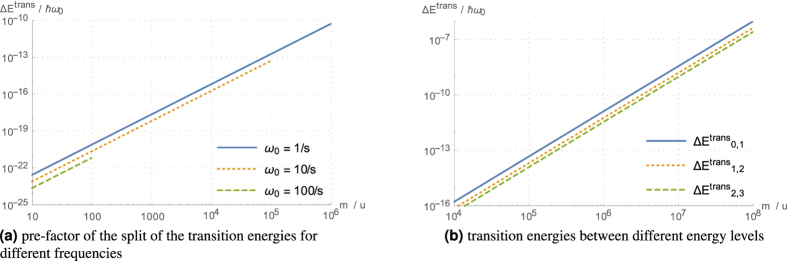
Dependence of the transition energies for wide wave-functions on the mass. The first plot shows the mass dependence of the pre-factor in equation (63) for different trap frequencies. The second plot shows the mass dependence for the actual transition energies between the first four energy levels for a trap frequency *ω*_0_ = 1 s^−1^. We used *σ* ≈ 7.0 × 10^−12^ m for silicon, as in the text. The plotted lines end for the mass value for which the wave-function width equals the radius *R* of the microsphere.

The resulting transition energies are


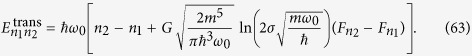



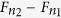
 is of the order of unity. The magnitude of the pre-factor for different trap frequencies, as well as the transition energies for a fixed frequency of 1 s^−1^ are plotted in Fig. 5. Since for the same trap frequencies the mass must be smaller in order to still be in the wide wave-function regime, the transition energies are several orders of magnitude below those for the intermediate regime.

There is also a “semi-wide” regime where the wave-function width is between *σ* and *R*. We omit the detailed discussion of this regime here. This has no effect on the qualitative results provided, although this regime could in principle be treated along the same lines. Experimentally, the narrow and intermediate regime are the most relevant for trapped microspheres. We mainly discussed the wide wave-function here for reasons of completeness, and because this is the situation at hand in experimental tests based on molecular interferometry.

## Gravitational dynamics of squeezed coherent Gaussian states

Inspired by the proposal by Yang *et al*.^18^, here we discuss the dynamical properties of a trapped microsphere that has been prepared in a squeezed ground state. A particular property of a harmonic potential is that a Gaussian wave packet remains Gaussian during its time evolution. This is because a Gaussian is fully determined by the first and second moments, 〈*x*〉, 〈*p*〉, 〈*x*^2^〉, 〈*p*^2^〉, and the correlation 〈*xp* + *px*〉, and the Schrödinger equation gives a closed system of equations for the same.

This property does not persist if a non-quadratic potential, such as our gravitational potential *V*_*g*_, is added to the Hamiltonian. However, since the potential *V*_*g*_ is usually weak compared to the harmonic trap potential, we can assume that the dynamics of an initially Gaussian wave packet are still approximately determined by the time evolution of the first and second moments^16^,^17^,^18^.

With the general Schrödinger equation (28) one gets for the first moments^16^


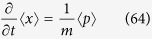



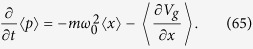


Since 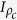 is an even function, and hence its derivative is odd, the expectation value of the derivative of *V*_*g*_ vanishes for any wave-function and any mass density function *ρ*_*c*_. Therefore, the time evolution of the first moments remains completely unchanged by self-gravitation, as one would intuitively expect, and in agreement with the Ehrenfest theorem.

For the second moments, first define the three-dimensional vector 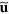 with components


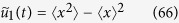



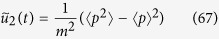



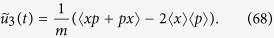


Then the Schrödinger equation (28) yields the following system of equations:


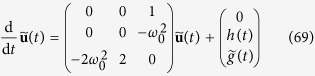


with


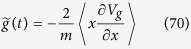



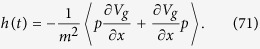


The function *h*(*t*) can be shown to equal (see Supplementary Information B)


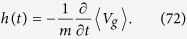


Redefining 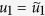, 
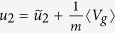
, 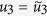, and


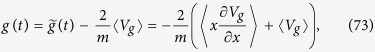


the system (69) then takes the form


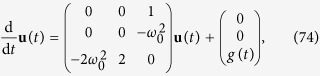


and is equivalent (given corresponding initial conditions) to the third order equation for *u*_1_


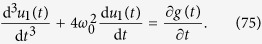


Up to this point, no restrictions have been imposed on the shape of the wave-function. Note, however, that *g*(*t*) depends on the wave-function. Hence, the right-hand-side is not a mere inhomogeneity but it renders the equation nonlinear, and in general the system (74) will not be closed. If now we assume that the wave-function is of Gaussian shape,


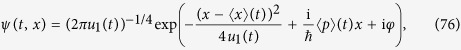


then the gravitational potential *V*_*g*_ is completely determined (for a given solution of the system of eqns (64) and (65) for the first moments) by the wave-function width *u*_1_(*t*), and so is the function *g*(*t*). We then obtain instead of equation (75) the closed equation


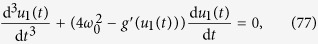


where the prime denotes the derivative by *u*_1_. In this case, the gravitational interaction acts like a wave-function width dependent change of the frequency of the internal oscillations of a Gaussian state.

Obviously, such internal oscillations appear only in the case of a squeezed state—for a coherent ground state, *u*_1_ would be a constant in time. Without the gravitational interaction, starting initially with a state whose width is *κ* times the width of the ground state, the solution of equation (77) is


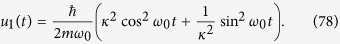


Hence, without gravity the width of the wave-function and 〈*x*〉 oscillate in phase. The gravitational interaction only affects the oscillation frequency of *u*_1_, and not that of 〈*x*〉, and therefore induces a de-phasing. This can, in principle, be observed experimentally.

In order to obtain quantitative results, we calculate the function *g*. Inserting the gravitational potential (27) and the wave-function (76) into (73) yields


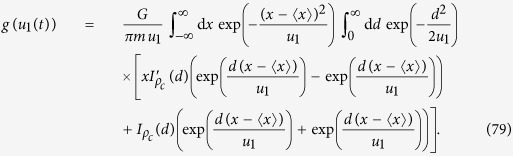


Introducing dimensionless variables, as in the previous section,


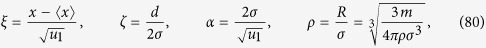


this can be rewritten as


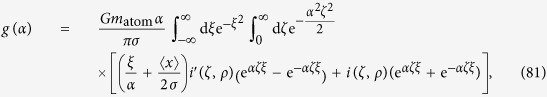


where 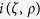 is defined as in the previous section, and 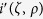 denotes the derivative by *ζ*. Evaluating the *ξ*-integral and taking the derivative leads to the desired function


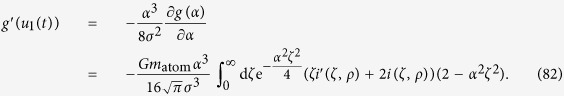


As before, we discuss the limits of a narrow and wide wave-function, and the intermediate regime.

### Narrow wave-functions

First we consider the limit *α* → ∞, corresponding to a narrow wave-function. We have


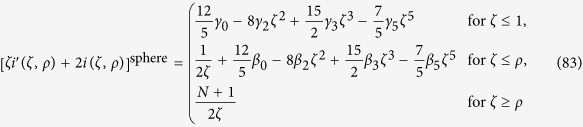


and


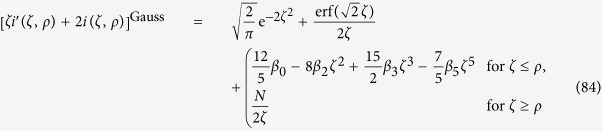



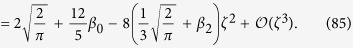


As before, we use that 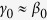, 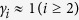, and 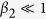 for 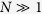 and 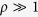. With this, to lowest order one simply obtains


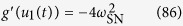


with the respective values (20) and (24) for the spherical and the Gaussian mass distributions.

Hence, we recover the result from Yang *et al*.^18^, that for a narrow wave-function the Schrödinger–Newton interaction yields a frequency shift to


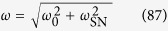


for the internal oscillations.

### Intermediate wave-functions

If *α* approaches values of the order of unity, we can split up the integral in a similar way as in the case of the energy spectrum above. Making use of 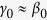, one obtains


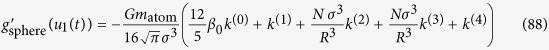


and


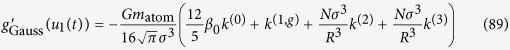


with


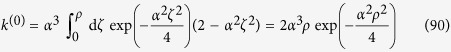



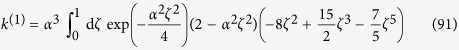



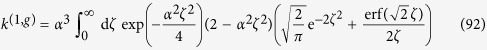



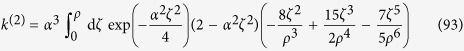



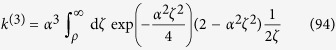



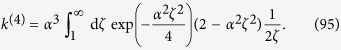


**Figure 6:**
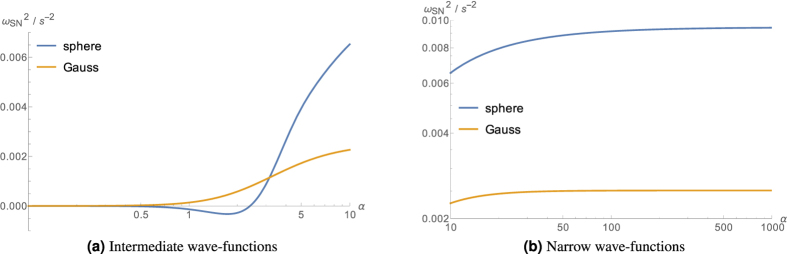
The plots show the dependence of the gravitational correction of the internal oscillation frequency in the regimes of narrow and intermediate wave-functions, respectively, with respect to the parameter *α*. Values are for silicon at 10 K.

For *ρ* ≫ 1 both *k*^(0)^ and *k*^(3)^ can be neglected. For *ρ* → ∞, 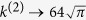, but since *k*^(2)^ is multiplied with the small pre-factor *Nσ*^3^/*R*^3^ it can be neglected in comparison to *k*^(1)^, *k*^(1,*g*)^, and *k*^(4)^ as well, just like 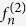 in the case of the energy spectrum. The resulting 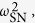 according to equation (86), is plotted as a function of the parameter *α* in Fig. 6, for both the spherical and the Gaussian mass distribution, in the regimes of narrow (*α* ≫ 1) and intermediate wave-functions (*α* ≈ 1). One can see from Fig. 6a that the values (20) and (24) are recovered in the limit *α* → ∞.

The *α*-dependence of 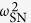 turns equation (77) into a nonlinear differential equation for the wave-function width. However, for finite values of *α*, 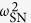 only becomes smaller compared to the narrow wave-function case. Therefore, in order to experimentally observe the frequency shift for the internal oscillations, the wave-function should be as narrow as possible, contrary to the energy spectrum, where we found the largest effect in the intermediate regime.

### Wide wave-functions

Finally, in the limit *α* → 0, i.e. for very wide wave-functions, the dominant contribution comes from *k*^(3)^, yielding


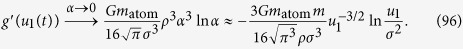


Inserting this result into (77) yields a nonlinear differential equation, whose solution gives the deviation from the behaviour without gravity. The effect is, however, much smaller for the wide wave-function than in the case of narrow wave-functions.

## Discussion

In this paper we provided a thorough survey of the effects of the gravitational self-interaction, described by the Schrödinger–Newton equation, on both the stationary states and the dynamics of a micron-sized sphere in a harmonic trap potential. We took the finite size of the system into account, as well as its crystalline substructure, and discussed the results for the different regimes of a wave-function that is wide, narrow, and comparable in width with the localisation of the nuclei in the crystal.

For the dynamics of a squeezed Gaussian state we recover the previous result^18^, that for a narrow state there is a frequency shift for the internal oscillations, and hence a de-phasing compared to the oscillations of the centre, 〈*x*〉, of the wave-function. The conclusion by Yang *et al*.^18^ was that for a silicon crystal at 10 K and a trap frequency of 2*π* × 10 s^−1^ a quality factor of 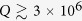 would be required for an experimental test of the Schrödinger–Newton effect.

Here, we could show that this result in the limit of a narrow wave-function is a best case scenario, in the sense that for a wider wave-function the de-phasing between internal and external oscillations only becomes smaller. We conclude from our considerations that for the given values^18^ a minimum mass of about 10^15^ atomic mass units is required. Below that mass, i.e. for 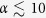, the amount of de-phasing drops considerably.

Contrary to this, we found that for the energy spectrum the Schrödinger–Newton effect is most pronounced in the regime of intermediate wave-functions. This is because the degenerate spectral lines at 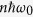, for a fixed *n*, are all shifted by the same amount in the narrow wave-function regime, while for wider wave-functions this degeneracy is removed, yielding a characteristic effect. The relative size of this effect is comparable to the dynamical frequency shift, providing a second possible basis for an experimental test of the Schrödinger–Newton equation. We propose a particular experiment based on this gravitational fine-structure elsewhere^19^.

It is also worth to remark that both effects, the dynamical and the spectral effect, scale proportional to the atomic mass and the inverse cubed localisation of the atoms. For elemental crystals, this scaling factor is maximal for osmium, although experimental requirements might necessitate a trade-off with other desirable material properties.

A situation that has not been considered here, but might be of relevance for experimental tests of the Schrödinger–Newton equation, is self-gravitation of a superposition of (a small number of) energy-eigenstates. A naive perturbative approach fails for times that are large compared to the oscillation period of the trap. Hence, alternative approximation schemes are necessary in order to describe these states, that are neither stationary nor Gaussian.

## Additional Information

**How to cite this article**: Großardt, A. *et al*. Effects of Newtonian gravitational self-interaction in harmonically trapped quantum systems. *Sci. Rep.* **6**, 30840; doi: 10.1038/srep30840 (2016).

## Supplementary Material

Supplementary Information
