# Supplementary material for: Effects of Newtonian gravitational self-interaction in harmonically trapped quantum systems
Source: Sci Rep. 2016 Aug 4;6:30840. doi: 10.1038/srep30840 (PMC4973281; doi:10.1038/srep30840)
Supplement: Supplementary Information [file srep30840-s1.pdf]

Supplementary Information to: Effects of Newtonian gravitational self-interaction in harmonically trapped quantum systems

André Großardt[1,2,*], James Bateman[3,4], Hendrik Ulbricht[3], and Angelo Bassi[1,2]

[1]Department of Physics, University of Trieste, 34151 Miramare-Trieste, Italy
[2]Istituto Nazionale di Fisica Nucleare, Sezione di Trieste, Via Valerio 2, 34127 Trieste, Italy
[3]School of Physics and Astronomy, University of Southampton, SO17 1BJ, United Kingdom
[4]Department of Physics, College of Science, Swansea University, Swansea SA2 8PP, United Kingdom
[*]andre.grossardt@ts.infn.it

SUPPLEMENTARY INFORMATION A: Axially symmetric stationary states

In the discussion in the paper we were, for reasons of simplicity, restricted to the one-dimensional Schrödinger–Newton equation. Realistic experimental scenarios may however require—for practical reasons—that the assumption of a strongly trapped wave-function in two dimensions must be given up. As a generalisation, here we discuss the axially symmetric situation of a microsphere in a trap with frequency ω_0 in x-direction, as before, but a finite frequency $\mu\,\omega_0$ in y- and z-direction. We further assume, that the system is in the ground state in y- and z-direction, such that the unperturbed state is

$$\psi_n^{(0)}(\mathbf{r}) = \frac{\sqrt{\mu}}{\sqrt{2^n\,n!}} \left(\frac{m\,\omega_0}{\pi\hbar}\right)^{3/4} \exp\left[-\frac{m\,\omega_0}{2\hbar}\left(x^2+\mu(y^2+z^2)\right)\right] H_n\left(\sqrt{\frac{m\,\omega_0}{\hbar}}\,x\right), \tag{S1}$$

with the Hermite polynomials as defined in equation (26b) of the paper.

In full analogy to the one-dimensional derivation, we obtain the energy correction

$$\begin{aligned}\Delta E_n = &-\frac{G\mu^2}{(2^n\,n!)^2}\left(\frac{m\,\omega_0}{\pi\hbar}\right)^3 \int \mathrm{d}^3r \int \mathrm{d}^3r'\, H_n\left(\sqrt{\frac{m\,\omega_0}{\hbar}}\,x\right)^2 H_n\left(\sqrt{\frac{m\,\omega_0}{\hbar}}\,x'\right)^2 \\ &\times \exp\left[-\frac{m\,\omega_0}{\hbar}\left(x^2+x'^2+\mu(y^2+y'^2+z^2+z'^2)\right)\right] I_{\rho_c}(|\mathbf{r}-\mathbf{r}'|)\,.\end{aligned} \tag{S2}$$

Again, we introduce dimensionless variables

$$\xi = \sqrt{\frac{m\,\omega_0}{\hbar}}\,x\,, \qquad s = \sqrt{\frac{m\,\omega_0}{\hbar}\,(y^2+z^2)}\,, \qquad \alpha = 2\,\sigma\sqrt{\frac{m\,\omega_0}{\hbar}}\,, \qquad \rho = \frac{R}{\sigma}\,, \tag{S3}$$

as well as

$$\zeta = \frac{|\mathbf{r}-\mathbf{r}'|}{2\,\sigma} = \frac{1}{\alpha}\sqrt{(\xi-\xi')^2+s^2+s'^2-2\,s\,s'\cos\varphi}\,. \tag{S4}$$

With this we get

$$\Delta E_n = -\frac{G\hbar\,m_{\text{atom}}}{4\,\sigma^3\,\omega_0}\,f_n(\alpha,\rho) \tag{S5a}$$

with

$$\begin{aligned}f_n(\alpha,\rho) = &\frac{2\,\alpha^2\,\mu^2}{(2^n\,n!)^2\,\pi^2}\int_{-\infty}^{\infty}\mathrm{d}\xi\int_{-\infty}^{\infty}\mathrm{d}\xi'\int_0^{\infty}\mathrm{d}s\int_0^{\infty}\mathrm{d}s'\int_0^{2\pi}\mathrm{d}\varphi \\ &\times s\,s'\,H_n(\xi)^2\,H_n(\xi')^2\exp\left(-\xi^2-\xi'^2-\mu\,s^2-\mu\,s'^2\right)\,i(\zeta,\rho)\,.\end{aligned} \tag{S5b}$$

The difference to the previous form in the one-dimensional case, eq. (29) in the paper, is only in the more complicated form, and μ-dependence, of the integral function f_n. This f_n can not be solved analytically any more. Values can, however, still be obtained by numerical integration.

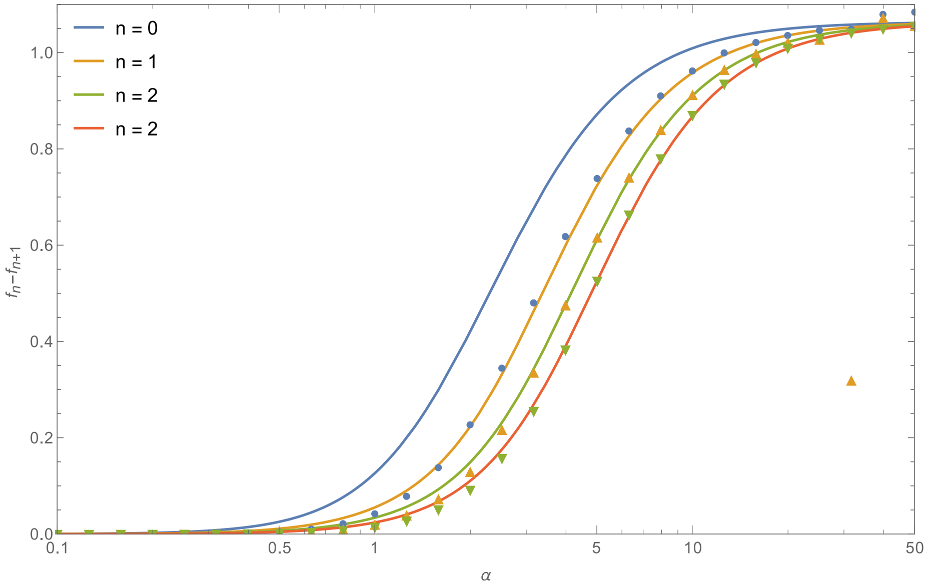

Figure S1. Comparison of the values for $f_n - f_{n+1}$ for the analytically obtained one-dimensional states (solid lines) and numerical results (data points) for the three-dimensional case with trap frequencies $\omega_x = \omega_0 = 2\omega_y = 2\omega_z$. Same colours belong to same n.

We are interested in transition energies in the intermediate regime for the Gaussian mass distribution, where we—following the discussion in the section about spectral effects in the intermediate regime in the paper—approximately take $\rho \to \infty$. Therefore we have

$$i(\zeta) \approx \frac{\operatorname{erf}\left(\sqrt{2}\,\zeta\right)}{2\,\zeta} \tag{S6}$$

where we neglected the constant term $\sim \beta_0$, because it does not contribute to transition energies, and neglected the terms $\sim \beta_k$ for $k \leq 2$ because they are small.

The integral in equation (S5b) can be further simplified for numerical evaluation by substituting $u = \exp(-\mu\, s^2)$, and accordingly for s'. We then get

$$f_n(\alpha) = \frac{1}{2}\left(\frac{\alpha}{2^n\, n!\, \pi}\right)^2 \int_{-\infty}^{\infty} \mathrm{d}\xi \int_{-\infty}^{\infty} \mathrm{d}\xi' \int_0^1 \mathrm{d}u \int_0^1 \mathrm{d}u' \int_0^{2\pi} \mathrm{d}\varphi \\ \times H_n\left(\xi\right)^2 H_n\left(\xi'\right)^2 \exp\left(-\xi^2 - \xi'^2\right) \frac{\operatorname{erf}\left(\sqrt{2}\,\zeta\right)}{2\,\zeta}\,, \tag{S7a}$$

with

$$\zeta = \frac{1}{\alpha}\sqrt{(\xi - \xi')^2 - \frac{\ln u}{\mu} - \frac{\ln u'}{\mu} - \frac{2}{\mu}\sqrt{\ln u \,\ln u'}\,\cos\varphi}\,. \tag{S7b}$$

Since the integrand is highly oscillating, one must carefully choose a convenient numerical integration method. We used the *Divonne* algorithm from the *Cuba* library[1].

In Fig. S1 the numerically obtained results $f_n - f_{n+1}$ for a value $\mu = 1/2$ are plotted. One can see that the effect discussed in the paper remains present also in this fully three-dimensional situation, qualitatively and from its order of magnitude. Interestingly, the numerical results suggest that for a trap frequency ratio of $2^k : 1 : 1$ the transition energies simply shift by k, i. e. the transition $0 \to 1$ corresponds to the transition $k \to k+1$ in the one-dimensional case, and so on. However, an analytical argument for this behaviour has yet to be found.

SUPPLEMENTARY INFORMATION B: Simplification of the function h(t)

We want to show that the function $h(t)$ defined in eq. (44c) of the paper can be written

$$h(t) = -\frac{1}{m^2}\left\langle p\,\frac{\partial V_g}{\partial x} + \frac{\partial V_g}{\partial x}\,p\right\rangle = -\frac{2}{m}\,\frac{\partial}{\partial t}\,\langle V_g\rangle\,. \tag{S8}$$

First note that with the probability current density,

$$j(x) = \frac{\mathrm{i}\hbar}{2m}\left(\psi(x)\,\frac{\partial\psi^*(x)}{\partial x} - \psi^*(x)\,\frac{\partial\psi(x)}{\partial x}\right)\,, \tag{S9}$$

we can write

$$\psi^*(x)\,\frac{\partial\psi(x)}{\partial x} = \frac{1}{2}\,\frac{\partial}{\partial x}\,|\psi(x)|^2 + \frac{\mathrm{i}m}{\hbar}\,j(x)\,. \tag{S10}$$

With this and the definition (22) in the paper for the gravitational potential, we can write

$$h(t) = -\frac{\mathrm{i}\hbar G}{m^2}\int\mathrm{d}x\int\mathrm{d}x'\left(|\psi(x)|^2\left|\psi(x')\right|^2\,\frac{\partial^2 I_{\rho_c}(|x-x'|)}{\partial x^2}\right. \tag{S11}$$

$$\left. +2\,\psi^*(x)\,\frac{\partial\psi(x)}{\partial x}\,\left|\psi(x')\right|^2\,\frac{\partial I_{\rho_c}(|x-x'|)}{\partial x}\right)$$

$$= -\frac{\mathrm{i}\hbar G}{m^2}\int\mathrm{d}x'\left|\psi(x')\right|^2\int\mathrm{d}x\left(|\psi(x)|^2\,\frac{\partial^2 I_{\rho_c}(|x-x'|)}{\partial x^2} + \frac{\partial\,|\psi(x)|^2}{\partial x}\,\frac{\partial I_{\rho_c}(|x-x'|)}{\partial x}\right) \tag{S12}$$

$$+\frac{2G}{m}\int\mathrm{d}x'\,\left|\psi(x')\right|^2\int\mathrm{d}x\,j(x)\,\frac{\partial I_{\rho_c}(|x-x'|)}{\partial x}$$

$$= -\frac{2G}{m}\int\mathrm{d}x'\,\left|\psi(x')\right|^2\int\mathrm{d}x\,\frac{\partial j(x)}{\partial x}\,I_{\rho_c}(\left|x-x'\right|) \tag{S13}$$

$$= \frac{2G}{m}\int\mathrm{d}x'\,\left|\psi(x')\right|^2\int\mathrm{d}x\,\frac{\partial\,|\psi(x)|^2}{\partial t}\,I_{\rho_c}(\left|x-x'\right|) \tag{S14}$$

$$= \frac{G}{m}\int\mathrm{d}x'\int\mathrm{d}x\,\frac{\partial}{\partial t}\left(\left|\psi(x')\right|^2|\psi(x)|^2\right)\,I_{\rho_c}(\left|x-x'\right|) \tag{S15}$$

$$= -\frac{1}{m}\,\frac{\partial}{\partial t}\,\langle V_g\rangle\,, \tag{S16}$$

where we made use of the continuity equation from (S13) to (S14).

References

1. Hahn, T. Cuba—a library for multidimensional numerical integration. *Comput. Phys. Commun.* **168**, 78–95 (2005). hep-ph/0404043.
